# A case of masked toxic adenoma in a patient with non-thyroidal illness

**DOI:** 10.1186/1472-6823-14-1

**Published:** 2014-01-02

**Authors:** Eun Ae Cho, Jee Hee Yoon, Hee Kyung Kim, Ho-Cheol Kang

**Affiliations:** 1 Department of Internal Medicine, Chonnam National University Medical School, Gwangju, South Korea; 2 Department of Internal Medicine, Chonnam National University Hwasun Hospital, Chonnam National University Medical School, 322 Seoyang-ro, Hwasun-eup, Hwasun-gun, Jeonnam 519-763, South Korea

**Keywords:** Thyroid, Non-thyroidal illness, Hyperthyroidism, Toxic adenoma

## Abstract

**Background:**

Non-thyroidal illness (NTI) refers to changes in thyroid hormone levels in critically ill patients in the absence of primary hypothalamic-pituitary-thyroid dysfunction, and these abnormalities usually resolve after clinical recovery. However, NTI can be accompanied by primary thyroid dysfunction. We report herein a case of a woman with NTI accompanied by primary hyperthyroidism.

**Case presentation:**

A 52-year-old female was admitted to the intensive care unit with heart failure and atrial fibrillation. She had a longstanding thyroid nodule, and a thyroid function test revealed low levels of triiodothyronine and free thyroxine as well as undetectable thyroid stimulating hormone (TSH). She was diagnosed with NTI, and her TSH level began to recover but not completely at discharge. The thyroid function test was repeated after 42 months to reveal primary hyperthyroidism, and a thyroid scan confirmed a toxic nodule.

**Conclusion:**

This case suggests that although NTI was diagnosed, primary hyperthyroidism should be considered as another possible diagnosis if TSH is undetectable. Thyroid function tests should be repeated after clinical recovery from acute illness.

## Background

Non-thyroidal illness (NTI) or euthyroid sick syndrome (ESS) is defined as a change in thyroid function during starvation or illness including a central reduction in thyroid stimulating hormone (TSH) secretion, decreased plasma triiodothyronine (T_3_) levels and decreased thyroxine (T_4_) and T_3_ binding in serum [1]. It is a relatively common syndrome, affecting about 70% of hospitalized patients [2] and may occur with virtually any illness [3]. Abnormalities in thyroid function can occur within hours of acute illness, and the magnitude of reduction in thyroid hormone levels correlates with disease severity and mortality. Serum T_3_ and free T_4_ levels are independent predictors of survival [4-6], and low levels of T_3_ are a poor prognostic factor of short- and long-term survival in patients with heart failure, acute myocardial infarction or acute stroke outside the intensive care unit (ICU) setting [7,8]. The pathophysiological mechanism responsible for NTI is complex and changes in the hypothalamic–pituitary–thyroid axis are the effect of various factors. Treatment of NTI with thyroid hormone is controversial, and abnormalities in thyroid function tests usually resolve after clinical recovery [3]. Primary thyroid dysfunction can be accompanied by NIT, and a follow-up thyroid function test is essential after recovery from illness to ascertain normalization. Here, we present a case of a woman with NTI who was later diagnosed with primary hyperthyroidism.

## Case presentation

**Figure 1:**
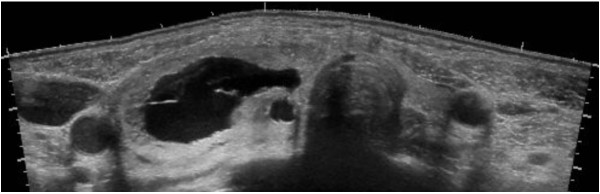
**Ultrasonographic findings.** Thyroid ultrasonogram demonstrates a 4.6 × 5.1 × 2.3 cm cystic dominant nodule in the lower portion of the right thyroid lobe and atrophy of the contralateral lobe.

A 52-year-old woman was admitted to the cardiology department ICU with generalized edema and orthopnea in July 2008. She had no significant medical history and was not taking any medications. An electrocardiogram revealed atrial fibrillation with a heart rate of 178 beats per minute. A chest X-ray detected pulmonary edema and cardiomegaly. A two-dimensional echocardiogram showed an ejection fraction of 57%, an enlarged left atrium without thrombi and grade 3 diastolic dysfunction. Diltiazem was used for heart rate control, but amiodarone or dopamine was not used during the hospitalization. Thyroid function tests were ordered as part of the atrial fibrillation evaluation. Serum free T_4_ level was 0.282 ng/dL (normal, 0.8–1.71 ng/dL), T_3_ was 0.307 ng/mL (normal, 0.6–1.6 ng/mL), and TSH was < 0.005 μIU/mL (normal, 0.4–4.8 μIU/mL). A physical examination revealed no apparent thyroid associated orbitopathy, but a large asymmetrical goiter, which was easily visible and palpable, was observed. A thyroid ultrasonogram demonstrated a 4.6 × 5.1 × 2.3 cm cystic dominant nodule in the lower portion of the right thyroid lobe and atrophy of the contralateral lobe (Figure 1). Five milliliters of serous, thin fluid was aspirated from the cyst. Aspiration cytopathology of the solid portion of the nodule was consistent with adenomatous goiter. An endocrine consultation was sought to evaluate the possibility of thyroid disease. She was diagnosed with NTI accompanying heart failure and a decision was made not to replace thyroid hormones but to follow-up on the thyroid function testing. Her symptoms resolved after 2 weeks, and she was subsequently discharged with a calcium channel blocker, an angiotensin receptor antagonist, a diuretic and aspirin. Thyroid function tests demonstrated a free T_4_ level of 0.860 ng/dL, and a TSH level of 0.017 μIU/mL at discharge.

**Figure 2:**
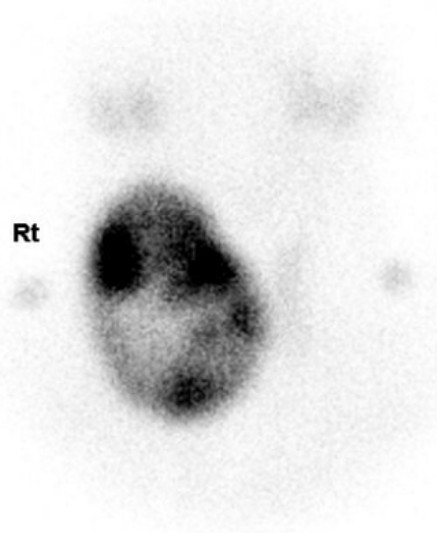
**Thyroid scan.** 99 m-Technetium pertechnetate thyroid scan reveals heterogeneously increased uptake in the large nodule of the right thyroid gland with decreased uptake by the remaining thyroid gland, suggesting a functioning nodule.

The thyroid function tests were not followed up until February 2012 (42 months) when she consulted the endocrinology department again for follow-up of the thyroid nodule. Her free T_4_ and T_3_ levels had increased to 5.30 ng/dL and 4.80 ng/mL, respectively, and TSH was < 0.005 μIU/mL. Anti-thyroid antibodies including anti-TPO antibody, anti-thyroglobulin antibody and TSH-binding inhibitory immunoglobulin were all negative. A follow-up thyroid ultrasonogram revealed an increase in the size of the previously observed nodule in the right lobe to 6.4 × 7.8 × 3.2 cm with contralateral lobe atrophy. A ^99m-^Technetium-pertechnetate thyroid scan demonstrated heterogeneous uptake in the large nodule of the right thyroid gland with no visibility in the remaining gland, suggesting a functioning toxic nodule (Figure 2). She refused an operation or radioactive iodine therapy; therefore, 10 mg methimazole twice per day was prescribed. Her free T_4_ and T_3_ levels normalised (0.873 ng/dL and 0.941 ng/mL, respectively) 2 weeks later, and TSH was 0.009 μIU/mL.

## Discussion

Our patient had symptoms and signs of heart failure and atrial fibrillation. She had an asymmetric goiter, her T_3_, free T_4_ and TSH levels all decreased significantly; thus, she was diagnosed with NTI. TSH began to increase at discharge, but none of the thyroid hormone levels normalized. There was a follow-up loss of the thyroid function tests until 42 months after discharge. Testing revealed primary hyperthyroidism and a thyroid scan confirmed a functioning toxic nodule. Thus, primary hyperthyroidism, particularly toxic adenoma, should be considered in patients with NTI who have an undetectable level of TSH and a huge nodule with an atrophic remaining thyroid gland.

NTI is a syndrome that reflects alterations in thyroid hormone levels during various illnesses [1]. Decreased total and free T_3_ levels with normal levels of TSH can be observed in the acute phase of critical illness (low T_3_ syndrome). Circulating T4 levels transiently rise during the acute phase of illness and normalize again when recovery follows quickly. However, patients who are severely ill and suffering from diseases that do not allow immediate recovery, present with reduced circulating total and free T4 concentrations. Reverse T_3_ increases due to impaired T_4_ conversion to T_3_ via peripheral deiodination. As the disease progresses, a dramatic fall in total T_4_ and T_3_ occurs (low T_4_ syndrome) [9], and about 50% of patients with NTI have decreased TSH levels, resulting from a reduction in thyroid releasing hormone secretion by the hypothalamus and indicating changes in hypothalamic–pituitary regulation [10]. Whether these changes are adaptive physiological mechanisms to conserve energy or consequences of the underlying illness is still a matter of debate [5,11]. However, treating NTI with thyroid hormones does not appear to be necessary, even though there is some controversy. For these reasons, routine thyroid function tests are not recommended in the intensive care setting unless a suspicion for thyroid dysfunction is based on history or a clinical evaluation. In the present case, a thyroid function test was ordered to evaluate atrial fibrillation and goiter.

The resolution of abnormal thyroid hormone levels after clinical recovery has been well documented. However, primary thyroid dysfunction can accompany NTI. TSH level may provide some clues for detecting underlying thyroid disease. Spencer et al. [12,13] reported that TSH can be low but detectable or high but < 20 μIU/mL in patients with NTI. The likelihood ratio for primary hyperthyroidism is 7.7 if TSH is undetectable, and the likelihood ratio is 11.1 for primary hypothyroidism if TSH > 20 μIU/mL [14]. Thus, TSH level should be considered in relation to the possibility of thyroid disease, and follow-up studies are mandatory, particularly if the value is not mildly abnormal. In our case, the patient had an undetectable level of TSH, and a thyroid ultrasonogram revealed a large cystic dominant nodule in the right thyroid lobe with atrophy of the opposite lobe. Although she was diagnosed with NTI, primary hyperthyroidism due to the toxic nodule should have been considered and a follow-up thyroid function test should have been performed after discharge.

Various drugs used in the hospital, particularly in the ICU, can alter thyroid function tests. Dopamine reduces serum TSH if used for a prolonged time [15]. Dobutamine, given in pharmacologic doses, also lowers TSH, even though TSH levels remain within the normal limits in subjects with a normal baseline TSH level [16]. Amiodarone is another drug that can cause alterations in thyroid function tests. Although most patients (>70%) on amiodarone remain euthyroid, the drug can lead to either amiodarone-induced hypothyroidism or amiodarone-induced thyrotoxicosis. High-dose glucocorticoids and octreotide also transiently suppress TSH, although central hypothyroidism does not appear to occur with these drugs [17,18]. Attention should be paid when interpreting thyroid function tests if any of these drugs have been used. Our patient never received any of these drugs.

A toxic nodule is a solitary, autonomously functioning thyroid nodule. The pathogenesis includes mutations in the TSH receptor leading to enhanced stimulation of thyroid follicular cell proliferation and function [19]. A thyroid nodule generally large enough to be palpable is present with suppressed TSH level, as in the present case. Patients initially have subclinical hyperthyroidism but when the adenoma grows to a significant size, frank hyperthyroidism develops, and elevated serum thyroid hormone levels accompany this condition [20]. Thyrotoxicosis is usually mild. A thyroid nodule appears on ultrasonography as a hypo-echogenic nodule with an atrophic thyroid gland. A thyroid scan is a definitive diagnostic test, demonstrating increased radioiodine uptake in the hyperfunctioning nodule and decreased uptake in the remaining gland. Radio-iodine ablation is usually the treatment of choice. Surgical resection of the adenoma or lobectomy to preserve thyroid function is another treatment option [1].

## Conclusion

NTI is a very common syndrome in the intensive care setting, and routine thyroid function testing is generally not recommended. However, if there is a high suspicion for underlying thyroid disease, a thyroid function test should be performed and interpreted with caution. Thyroid function tests should be repeated after recovery from acute illness to ascertain euthyroid status. An evaluation for primary thyroid disease is essential, particularly when TSH is undetectable or >20 μIU/mL.

### Consent

Written informed consent was obtained from the patient for publication of this case report and any accompanying images. A copy of the written consent is available for review by the Editor of this journal.

## Competing interests

No potential conflict of interest relevant to this article was reported.

## Authors’ contributions

EAC – 1^st^ author of case report. She involved in writing 1^st^ draft. JHY – contributed by revision it critically for intellectual consent. HKK – diagnosed and treated the patient, and analyzed current literature on the topic to format a discussion, conclusion of the presented case. HCK – consultant physician overseeing the case of this patient. He had final approval of the case report to be submitted. All authors read and approved the final manuscript.

## Disclosure statement

The authors have nothing to disclose.

## Pre-publication history

The pre-publication history for this paper can be accessed here:

http://www.biomedcentral.com/1472-6823/14/1/prepub

## References

[B1] Melmed SPK Larsen PR Kronenberg HM Williams Textbook of Endocrinology 2011 12 Philadelphia, PA: Saunders Elsevier 327 405

[B2] Bermudez F Surks MI Oppenheimer JH High incidence of decreased serum triiodothyronine concentration in patients with nonthyroidal disease J Clin Endocrinol Metab 1975 41 1 27 40 10.1210/jcem-41-1-27 807593

[B3] Adler SM Wartofsky L The nonthyroidal illness syndrome Endocrinol Metab Clin North Am 2007 36 3 657 672 vi 10.1016/j.ecl.2007.04.007 17673123

[B4] De Marinis L Mancini A Masala R Torlontano M Sandric S Barbarino A Evaluation of pituitary-thyroid axis response to acute myocardial infarction J Endocrinol Invest 1985 8 6 507 511 3833895 10.1007/BF03348548

[B5] De Groot LJ Dangerous dogmas in medicine: the nonthyroidal illness syndrome J Clin Endocrinol Metab 1999 84 1 151 164 10.1210/jc.84.1.151 9920076

[B6] Ray DC Drummond GB Wilkinson E Beckett GJ Relationship of admission thyroid function tests to outcome in critical illness Anaesthesia 1995 50 12 1022 1025 10.1111/j.1365-2044.1995.tb05943.x 8546279

[B7] Iervasi G Pingitore A Landi P Raciti M Ripoli A Scarlattini M L'Abbate A Donato L Low-T3 syndrome: a strong prognostic predictor of death in patients with heart disease Circulation 2003 107 5 708 713 10.1161/01.CIR.0000048124.64204.3F 12578873

[B8] Iglesias P Munoz A Prado F Guerrero MT Macias MC Ridruejo E Tajada P Diez JJ Alterations in thyroid function tests in aged hospitalized patients: prevalence, aetiology and clinical outcome Clin Endocrinol (Oxf) 2009 70 6 961 967 10.1111/j.1365-2265.2008.03421.x 18793343

[B9] Mebis L Van den Berghe G Thyroid axis function and dysfunction in critical illness Best Pract Res Clin Endocrinol Metab 2011 25 5 745 757 10.1016/j.beem.2011.03.002 21925075

[B10] Plikat K Langgartner J Buettner R Bollheimer LC Woenckhaus U Scholmerich J Wrede CE Frequency and outcome of patients with nonthyroidal illness syndrome in a medical intensive care unit Metabolism 2007 56 2 239 244 10.1016/j.metabol.2006.09.020 17224339

[B11] Wartofsky L Burman KD Ringel MD Trading one “dangerous dogma” for another? Thyroid hormone treatment of the “euthyroid sick syndrome” J Clin Endocrinol Metab 1999 84 5 1759 1760 10.1210/jc.84.5.1759 10323414

[B12] Spencer C Eigen A Shen D Duda M Qualls S Weiss S Nicoloff J Specificity of sensitive assays of thyrotropin (TSH) used to screen for thyroid disease in hospitalized patients Clin Chem 1987 33 8 1391 1396 3301067

[B13] Spencer CA LoPresti JS Patel A Guttler RB Eigen A Shen D Gray D Nicoloff JT Applications of a new chemiluminometric thyrotropin assay to subnormal measurement J Clin Endocrinol Metab 1990 70 2 453 460 10.1210/jcem-70-2-453 2105333

[B14] Attia J Margetts P Guyatt G Diagnosis of thyroid disease in hospitalized patients: a systematic review Arch Intern Med 1999 159 7 658 665 10.1001/archinte.159.7.658 10218744

[B15] Kaptein EM Spencer CA Kamiel MB Nicoloff JT Prolonged dopamine administration and thyroid hormone economy in normal and critically ill subjects J Clin Endocrinol Metab 1980 51 2 387 393 10.1210/jcem-51-2-387 7400302

[B16] Lee E Chen P Rao H Lee J Burmeister LA Effect of acute high dose dobutamine administration on serum thyrotrophin (TSH) Clin Endocrinol (Oxf) 1999 50 4 487 492 10.1046/j.1365-2265.1999.00678.x 10468908

[B17] Nicoloff JT Fisher DA Appleman MD Jr The role of glucocorticoids in the regulation of thyroid function in man J Clin Invest 1970 49 10 1922 1929 10.1172/JCI106411 4990073 PMC322682

[B18] Colao A Merola B Ferone D Marzullo P Cerbone G Longobardi S Di Somma C Lombardi G Acute and chronic effects of octreotide on thyroid axis in growth hormone-secreting and clinically non-functioning pituitary adenomas Eur J Endocrinol 1995 133 2 189 194 10.1530/eje.0.1330189 7655643

[B19] Parma J Duprez L Van Sande J Cochaux P Gervy C Mockel J Dumont J Vassart G Somatic mutations in the thyrotropin receptor gene cause hyperfunctioning thyroid adenomas Nature 1993 365 6447 649 651 10.1038/365649a0 8413627

[B20] Hegedus L Bonnema SJ Bennedbaek FN Management of simple nodular goiter: current status and future perspectives Endocr Rev 2003 24 1 102 132 10.1210/er.2002-0016 12588812

